# An Alleged New Treatment for Tuberculosis

**Published:** 1896-04

**Authors:** 


### EDITORIAL DEPARTMENT.

F. E. DANIEL, M. D., Editor.
S. E. HUDSON, M. D., Managing Editor.
A. J. SMITH. M. D., Galveston, Associate Editor.

EDITORIAL STAFF:

PROF. J. E. THOMPSON, M. D., Texas Medical College, Galveston; Surgery.
PROF. WM. KEILLER, M. D., Texas Medical College, Galveston; Obstetrics and Gynecology.
PROF. DAVID CERNA, M. D., Texas Medical College, Galveston; Therapeutics.
PROF. A. J. SMITH, M. D., Texas Medical College, Galveston; Medicine.
DR. R. H. L. BIBB, Saltillo, Mexico; Foreign Correspondent.

Official organ of the West Texas Medical Association, the Houston District Medical Association, the Austin District Medical Society, the Galveston County Medical Society, and several others.

### AN ALLEGED NEW TREATMENT FOR TUBERCULOSIS.

In the light of modern therapeutics it may properly be said, indeed, that it matters not whether the tubercle bacilli be the direct causative agents of pulmonary phthisis, as first enunciated by Koch and generally accepted now as the correct view, or whether these micro-organisms be merely the resultants of the disease, as held by some writers; the main object to the treatment of the disorder seems to be absolutely logical; that is, to get rid of the bacilli. This, at all events, has been the chief tendency in the present *fin de ciecle* clinical investigation. And in looking over the immense literature on the subject in recent years, it seems that, in the rational treatment of pulmonary tuberculosis, the most satisfactory results have been obtained, not by methods to bring about the destruction of the tubercle bacilli, but by measures tending to so prepare the field as to make it impossible for the specific germs to develop or grow.

But the whole history of the subject is one continuous sad story of disastrious failures, defeated ambitions, blasted hopes, with here and there the annihilation of a brilliant career through the actual loss of a precious life, and all in the endeavor to solve the most difficult problem, perhaps, of medical science. For neither the sulphuretted hydrogen of Burgeon, nor the tuberculine of Koch, nor the tuberculocedine of Klebs, nor the cantharidine of Liebreich, nor the chloride of zinc of Lannelongue, nor a host of other remedies proposed by various investigators, seem to have accomplished much toward staying the fatal stroke of the dread malady. Pulmonary phthisis, in spite of all earnest and careful research, continues to reign supreme as the greatest, almost invincible, enemy of the human race!

And yet, when we remember that many cases of well developed tubercular disease get well under a good, carefully regulated nourishing diet, a diet directed mainly to sustain the nutritive forces of the body; when the fact is not lost sight of that often patients recover under hygienic surroundings, *without treatment*, and that, again, many cases are restored to good health *in spite of treatment*, we are indeed strengthened in the belief that the terrible scourge under consideration needs really no special medication, no specific management. In the treatment of tuberculosis, as in that of most other cases of disease, the application of general principles, when the object of securing a proper nutrition, by intelligently aiding the course of nature, should be the rule, not the exception. It follows, therefore, that *no exclusive plan of management* can be expected to produce always the same, if any, beneficial results. In support of our assertion the history of the complete failure met with by every specific remedy proposed in recent years, stands out in bold relief!

And because tuberculosis continues to defeat all efforts in the search of an effective remedial agent, shall medical science give up in despair? Shall the field be abandoned entirely to the enemy? It can not, it must not be. This brings to our mind the eloquent words of our great Da Costa who, in his brilliant essay on "Harvey and his Discovery," says: "The indisposition to receive the new is, in fact, disappearing from among us very fast, and the period of probation for valuable discoveries is becoming shorter and shorter. What is true is sure of speedy acceptance; and if, after a lapse of years, not now a long one, a fact generally disseminated or a system keenly discussed is rejected by those qualified to judge, it is because it is false. There is for matters intellectual and for facts scientific a statute of limitation framed up by the cultivated; that which is outside the statute becomes pseudo-science and unfounded assertion. We find this in all the so-called exclusive systems in medicine. We know that they are not true, because they have been so long before us, and are so universally rejected by those best fitted to pronounce judgment. If true, like the startling innovation of Harvey, they would long since have made their way in the profession. One generation might have ignored or opposed them; but the young of that generation would have discussed them and leaned towards them. Did not Jenner live to see vaccination ardently approved of in the four quarters of the globe? Have not anæsthetics, has not the hypodermic method, rapidly overcome every opposition? Were not the great discoveries of Bernard—of all of our day the one whose work Harvey would most have delighted in—were they long before us before they were enthusiastically incorporated in our science? No; so eager are we after the truth; so keenly conscious that we have to contend with a foe from whom we can expect nothing, and with whom we can entertain no conditions of peace; so bound to welcome whatever seems likely to aid us in the struggle, that we can not, we dare not, long reject anything that promises success. Where life is concerned, where friends, brothers, children, have to depend on our knowledge and the means we can command, prejudice is hushed, lukewarmness can not enter; a remedy taken out of the street would soon work its way to universal favor. No; the banner that medicine rallies her sons under is the banner of progress, eagerly upheld by stalwart arms and enthusiastic hearts in search only of the truth."

Dr. Cyrus Edson, of New York, as the result of certain original investigations, has quite recently proposed a new treatment for pulmonary tuberculosis; and the profession, as well as the laity, will, of course, examine the alleged discovery in the hope of finding something that may be of service to suffering humanity. What is this new treatment? Let us see.

According to the observations of Brieger, Merck, Salkowski, and others, phenol is a normal condition of the urine, that substance having been detected in the urine of the horse and the cow, as well as in that of man. Again, the fact has been ascertained that during disease, especially in febrile conditions, the percentage of phenol in the urine is increased to a considerable extent. Moreover, Edson believes that the high fever under such circumstances is probably the result of poisoning of the nerve centers by phenol the increased secretion of which, during disease, is but an evidence of the *vis medicatrix naturæ*. This is mind, the author has thought that "if nature herself provides phenol during disease, then it cannot be possible she will not tolerate the administration of the agent in effective dosages." The success obtained by various clinical observers in the creosote treatment of tuberculosis, Edson ascribes to the action of phenol contained in that remedy; for it must not be forgotten that creosote and phenol are not identical substances, the latter being only a constituent of the former medicament.

The question to be solved, then, would be to determine a method by which sufficiently large doses of phenol could be administered without producing deleterious effects. Working on this point, Edson (so he alleges) has finally succeeded in producing a solution of phenol with pilocarpine.

Physiological studies of the actions of this latter drug, pilocarpine, have shown that the alkaloid is a powerful diaphoretic, causing the separation of large amounts of water from the blood; that is, an expectorant, and a powerful stimulant of secretion. But the chief purpose of Edson in bringing about a combination of the two madicaments was based on the belief that pilocarpine can induce leucocytosis and marked glandular activity. Be all this as it may, that author claims that the fluid produced is a chemically pure solution of pilocarpine-phenyl-hydroxide to which the common name of *Aseptolin* has been applied; that, in fact, the fluid prepared in his laboratory "is a hydrophenol, containing a definite amount of the new pilocarpine compound." *Aseptolin* occurs in the form of a colorless liquid, strongly refracting light, and having the characteristic odor and taste of phenol. It causes a sharp, burning pain when injected hypodermically, but not so severe as that caused by solutions of corrosive sublimate. Edson states: "Though I have given over one thousand injections, and some of them very large ones, viz: single injections of 350 minims each, I have not seen a single abscess resulting therefrom, and nodulation in only two cases; one of these was on my own person. No reaction, such as follows the administration of tuberculine, is observed after the injection of properly prepared pilocarpine-phenyl-hydroxide solution, nor is there any visible physiological action following an injection of 250 minims, given to a man weighing 150 pounds, except that the urine passed subsequently reacted strongly to tests made to ascertain the presence of phenol, and traces of phenol were noted in the condensed vapor of the breath, and in the contents of the stomach drawn off through the œsophageal tube within three hours of injection."

To begin with, the author recommends a daily dose of from 50 to 70 minims, to be increased 10 minims per day until an amount of from 100 to 120 minims is reached. The dose is to be injected subcutaneously in the abdominal walls, either daily or every other day, according to the exigencies of the individual case. In "order to assist in cleaning up the larynx and bronchi of infective material contained in their secretions," the author also employs inhalations by means of sprays from ethereal solutions of iodoform of the strength of 10 per cent. In some cases these inhalations may be preceded to advantage, for a few times, by carbolic acid sprays.

The same method of treatment for tuberculosis, is recommended by Edson in malarial disease, and in septicæmia.

It appears, however, that *aseptolin* is not thrust upon the medical profession as a specific remedy. The usefulness of the new medicament proposed remains to be confirmed by future experience. But the newspaper notoriety given Edson's alleged discovery, a circumstance which savors of quackery, is deeply to be regretted, for that alone is sufficient to prejudice the honest, regular profession against the advertised "new treatment of consumption." Although Edson has published his observations in the matter in reputable medical journals, the undue notoriety given the "new cure" through the newspapers is a method highly to be condemned in professional circles. The newspapers are certainly not the proper channels to communicate an alleged new discovery in medical science, much less when accompanied with the spirit of secresy employed in Edson's case. For in this manner the new discovery (?) is placed on the level of quack nostrums. And as a recent editorial writer has so well expressed: "Nostrums for consumption are probably the best paying of all nostrums during the few years that their vogue lasts. Necessarily their flourishing period is restricted, for they soon cease to be 'new,' and cannot long continue to claim 'cures.' They die out with their victims in six years at most; or, at all events, unless their names and allegations of virtues are modified, they cease to be profitable. Usually they are still lauded as 'preventatives,' or sold as 'cough remedies,' but as 'consumption cures' they have become, as our German friends say, '*ausgespielt*.' "

But all in all, and in the light of calm and dispassionate judgment, further trials in the proposed new method in the treatment of a disease that continues to put the highest skill in medical thought and research to the severest tests, apparently in vain,—further trials of the proposed new method, we repeat, seem to be justifiable and to command some consideration. Although, in point of fact, we cannot understand how small doses of pilocarpine, such as 1-100, or even 1-50 of a grain, as advised to be taken daily, are expected to produce a glandular activity, when we know that $\frac{1}{8}$ of a grain is about the minimum dose of the alkaloid to cause any physiological effects. Again, in a disease in which all the powers appear to be so markedly depressed, it seems absurd to even suppose that large doses of a drug like phenol can do good in such cases, when all high authorities, such as Wood, Brunton and others, have shown by careful experimentation that this agent is a powerful poison to all higher tissues, especially to the central nervous system.

What the outcome will be we can not, we dare not predict. But would, indeed, that Edson's method could accomplish half as much in the treatment of tuberculosis as we have reason to believe the "weird and wonderful" discovery of Rontgen will in the wide realms of medical and surgical diagnosis. D. C.

